# Vitamin D deficiency and diabetic retinopathy risk in patients with newly diagnosed type 2 diabetes mellitus: a retrospective analysis

**DOI:** 10.3389/fnut.2025.1614287

**Published:** 2025-05-30

**Authors:** Kuo-Chuan Hung, Li-Chen Chang, Ying-Jen Chang, Chun-Ning Ho, Jheng-Yan Wu, Wei-Cheng Liu, I-Wen Chen

**Affiliations:** ^1^Department of Anesthesiology, Chi Mei Medical Center, Tainan, Taiwan; ^2^School of Medicine, College of Medicine, National Sun Yat-sen University, Kaohsiung, Taiwan; ^3^Department of Anesthesiology, E-Da Hospital, I-Shou University, Kaohsiung, Taiwan; ^4^Department of Recreation and Health-Care Management, College of Recreation and Health Management, Chia Nan University of Pharmacy and Science, Tainan, Taiwan; ^5^Department of Nutrition, Chi Mei Medical Center, Tainan, Taiwan; ^6^Department of Occupational Therapy, Shu-Zen Junior College of Medicine and Management, Kaohsiung, Taiwan; ^7^Department of Anesthesiology, Chi Mei Medical Center, Liouying, Tainan, Taiwan

**Keywords:** vitamin D deficiency, diabetic retinopathy, type 2 diabetes mellitus, propensity score matching, risk factors

## Abstract

**Background:**

Vitamin D deficiency (VDD) has emerged as a potential contributor to diabetic complications. This study aimed to investigate the association between VDD at the time of type 2 diabetes mellitus (T2DM) diagnosis and subsequent risk of developing diabetic retinopathy (DR).

**Methods:**

This retrospective cohort study used data from the TriNetX Research Network to analyze adult patients newly diagnosed with T2DM between January 2020 and December 2022. The patients were classified as vitamin D-deficient (<20 ng/mL) or sufficient (≥30 ng/mL). After 1:1 propensity score matching, 10,651 patients were included in each group. The primary outcome was the risk of DR within 3 years of T2DM diagnosis. The secondary outcomes included hospitalization, emergency department visits, pneumonia, and all-cause mortality. An exploratory analysis was also conducted to examine outcomes in patients with vitamin D insufficiency (20–30 ng/mL) compared to the sufficient group.

**Results:**

At the 3-year follow-up, VDD was significantly associated with an increased risk of DR [hazard ratio (HR) 1.45, 95% confidence interval (CI) 1.17–1.80, *p* < 0.001], hospitalization (HR 1.23, 95% CI 1.17–1.29, *p* < 0.001), emergency department visits (HR 1.17, 95% CI 1.11–1.24, *p* < 0.001), pneumonia (HR 1.18, 95% CI 1.07–1.31, *p* = 0.001), and mortality (HR 1.51, 95% CI 1.36–1.67, *p* < 0.001). Sex-stratified analysis revealed that the association between VDD and DR was significant among female patients (HR 1.41, 95% CI 1.07–1.86, *p* = 0.015) but not among males. Exploratory analysis showed that vitamin D insufficiency (20–30 ng/mL) was not associated with increased DR risk, suggesting a threshold effect.

**Conclusion:**

In newly diagnosed T2DM patients, VDD was independently associated with increased risks of DR and other adverse outcomes, particularly in females. The observed threshold effect suggests that maintaining vitamin D levels above the deficiency threshold may be sufficient to mitigate DR risk. Assessment of vitamin D status may be valuable for risk stratification in newly diagnosed T2DM, and addressing VDD may represent a modifiable risk factor for improving outcomes.

## 1 Introduction

Diabetic retinopathy (DR) is one of the most prevalent microvascular complications of diabetes mellitus (DM), and remains a leading cause of preventable blindness worldwide. With the steadily increasing global prevalence of type 2 diabetes mellitus (T2DM), identifying modifiable risk factors for DR has become increasingly important for developing effective preventive strategies and improving patient outcomes. Vitamin D deficiency (VDD) has emerged as a potential contributor to diabetic complications owing to its effects on inflammation, oxidative stress, and vascular function (1–3). The active form of vitamin D, 1,25-dihydroxyvitamin D3, exerts its biological effects through vitamin D receptors, which are widely expressed in retinal tissues including the retinal pigment epithelium, photoreceptors, and retinal vasculature (4). This specific localization in ocular structures provides a direct biological rationale for investigating the role of vitamin D in retinopathy. Growing evidence suggests that vitamin D may protect against retinal damage by inhibiting inflammation, reducing oxidative stress, and improving microvascular function (2, 5).

A recent systematic review and meta-analysis by Petrea et al. (6) analyzed data from 20 studies involving 22,408 participants and found a significant association between lower vitamin D levels and increased risk of DR [odds ratio (OR): 1.17]. Their analysis also revealed lower mean serum vitamin D levels in individuals with DR than in those without DR, with progressively declining levels across DR severity stages. However, most studies included (7–9) have been limited by their cross-sectional design, small sample sizes, and inadequate control for confounding factors. A 5-year prospective study by Herrmann et al. (10), which included 9,524 participants, found that lower serum 25(OH)D levels were associated with an increased risk of macrovascular and microvascular complications, including retinopathy, requiring laser therapy. However, their analysis involved patients with varying durations of diabetes and focused only on advanced DR (10). Moreover, vitamin D status was not assessed at the time of T2DM diagnosis. These limitations highlight the need for longitudinal studies in newly diagnosed patients.

We hypothesized that VDD at T2DM diagnosis is associated with an increased risk of DR and other adverse outcomes. This study aimed to investigate the association between VDD at the time of T2DM diagnosis and the subsequent risk of developing DR over a 3-year follow-up period. Additionally, we examined important secondary outcomes, including hospitalization, emergency department visits, pneumonia, and all-cause mortality, to provide a more comprehensive understanding of the potential impact of vitamin D status on overall health outcomes in newly diagnosed T2DM patients.

## 2 Methods

### 2.1 Data sources and ethical statement

This retrospective cohort study was conducted using data from the TriNetX Research Network, a global health research platform that aggregates de-identified electronic medical records from participating healthcare organizations located primarily in the United States (11–14). The network included data from inpatient, outpatient, and specialty care settings, allowing for longitudinal follow-up and detailed clinical profiling. All data were de-identified in compliance with the Health Insurance Portability and Accountability Act (HIPAA) and met the requirements of the U. S. Federal Policy for the Protection of Human Subjects. The study protocol was reviewed and approved by the review board of Chi Mei Medical Center (IRB Number: 11402-E02), and the requirement for informed consent was waived due to the use of de-identified data and minimal risk to patients.

### 2.2 Study design and patient selection

We included adult patients (≥18 years old) newly diagnosed with T2DM between January 1, 2020, and December 31, 2022. The index date was defined as the date of the first T2DM diagnosis (ICD-10-CM codes: E11). Eligible patients were required to have at least one recorded 25-hydroxyvitamin D [25(OH)D] level within 6 weeks prior to the index date. Patients with serum vitamin D levels <20 ng/mL were categorized into the VDD (VDD) group (15, 16), while those with levels ≥30 ng/mL were included in the control group. To minimize misclassification, we excluded patients in the VDD group who had any vitamin D measurement >20 ng/mL, and those in the control group if any measurement was <30 ng/mL at any time point.

To ensure a temporal relationship between baseline vitamin D status and incident DR, we excluded patients with any diagnosis of DR prior to the index date (17). In addition, to reduce potential misclassification or confounding of ocular outcomes, we excluded patients with conditions that may mimic or influence retinal pathology, such as pregnancy, cataracts, vitreous hemorrhage, age-related macular degeneration, glaucoma, retinal vascular occlusions, or hypertensive retinopathy, if diagnosed within 1 year before or 3 years after the index date. These exclusion windows were selected to enhance diagnostic specificity rather than to define disease latency.

### 2.3 Data collection

Baseline characteristics and comorbidities were extracted from the 2-year period before the index date. The collected variables included age, sex, race, essential hypertension, obesity, hyperlipidemia, neoplasms, chronic kidney disease (CKD), ischemic heart disease, liver disease, nicotine dependence, heart failure, chronic obstructive pulmonary disease, cerebrovascular disease, malnutrition, and alcohol-related disorders. Laboratory values were collected where available, including hemoglobin, albumin, and body mass index (BMI). For subgroup categorization, hemoglobin ≥12 g/dL, albumin ≥3.5 g/dL, and BMI ≥30 kg/m^2^ were used as clinical cutoffs. In addition, the use of common medications, such as antilipemic agents, ACE inhibitors, and angiotensin II receptor blockers, was recorded. Because all data in TriNetX were collected prior to the index date, anti-diabetic medication use was not included in the primary analysis, as such prescriptions may not have been initiated for diabetes management in patients newly diagnosed with T2DM, or the information may be incomplete.

### 2.4 Propensity score matching

To control for confounding factors, patients in the VDD and control groups were matched using 1:1 greedy nearest-neighbor propensity score matching. Matching was based on all baseline variables described above to ensure a balanced comparison between groups. TriNetX does not perform automatic imputation for missing data. Instead, a complete-case analysis approach was employed, whereby only patients with non-missing values for the covariates used in the propensity score model were included in the matching process. To evaluate the quality of propensity score matching, we generated propensity score density function plots to compare the distribution of propensity scores in both groups before and after matching. The density functions were plotted as a percentage of cohort against propensity score values (0–1), with Cohort 1 representing the VDD group and Cohort 2 representing the control group. Successful matching was indicated by overlapping density functions in the post-matching plot, confirming the balanced distribution of baseline characteristics.

### 2.5 Outcomes

The primary outcome was the incidence of DR within 3 years of the index date. The secondary outcomes included hospitalization, emergency department (ED) visits, pneumonia, and all-cause mortality during the same follow-up period. Additionally, to assess short-term associations, the 1-year incidence of these outcomes was evaluated.

### 2.6 Sensitivity analyses

Three sensitivity analyses were performed. First, to reduce potential survival bias, we restricted the cohort to patients who survived the entire 3-year follow-up period and reassessed the primary and secondary outcomes. Second, because antidiabetic medication use could not be evaluated in the primary analysis, we conducted a sensitivity analysis including all patients with T2DM, regardless of whether it was their initial diagnosis. In this cohort, we performed propensity score matching based on the same baseline variables as the primary analysis and included antidiabetic medication use to further assess the robustness of the association between vitamin D status and clinical outcomes. In addition, we performed a third sensitivity analysis (Model III) restricted to patients aged ≥50 years, a population with a higher baseline risk for T2DM and related complications.

### 2.7 Subgroup analysis

Subgroup analyses were conducted to assess whether the association between VDD and 3-year outcomes differed according to sex. Propensity score matching was repeated separately for male and female patients using the same variables and approach as in the primary analysis to ensure comparability within each subgroup.

### 2.8 Statistical analysis

Continuous variables were summarized using means and standard deviations, whereas categorical variables were described using frequencies and percentages. Baseline characteristics were compared using standardized mean differences (SMD), with an SMD <0.1 indicating a good balance between groups. Time-to-event outcomes, such as DR and mortality, were analyzed using Kaplan–Meier curves and compared using the log-rank test. Hazard ratios (HR) and 95% confidence intervals (CI) were calculated using Cox proportional hazards models. All statistical analyses were performed within the TriNetX platform, and a two-tailed *p*-value <0.05 was considered statistically significant.

In an additional exploratory analysis, we examined the outcomes of interest in patients with vitamin D insufficiency, defined as 25(OH)D levels of 20–30 ng/mL. This analysis aimed to explore whether intermediate vitamin D levels were associated with increased risks compared with sufficiency.

## 3 Results

### 3.1 Patient selection and baseline characteristics

**Figure 1:**
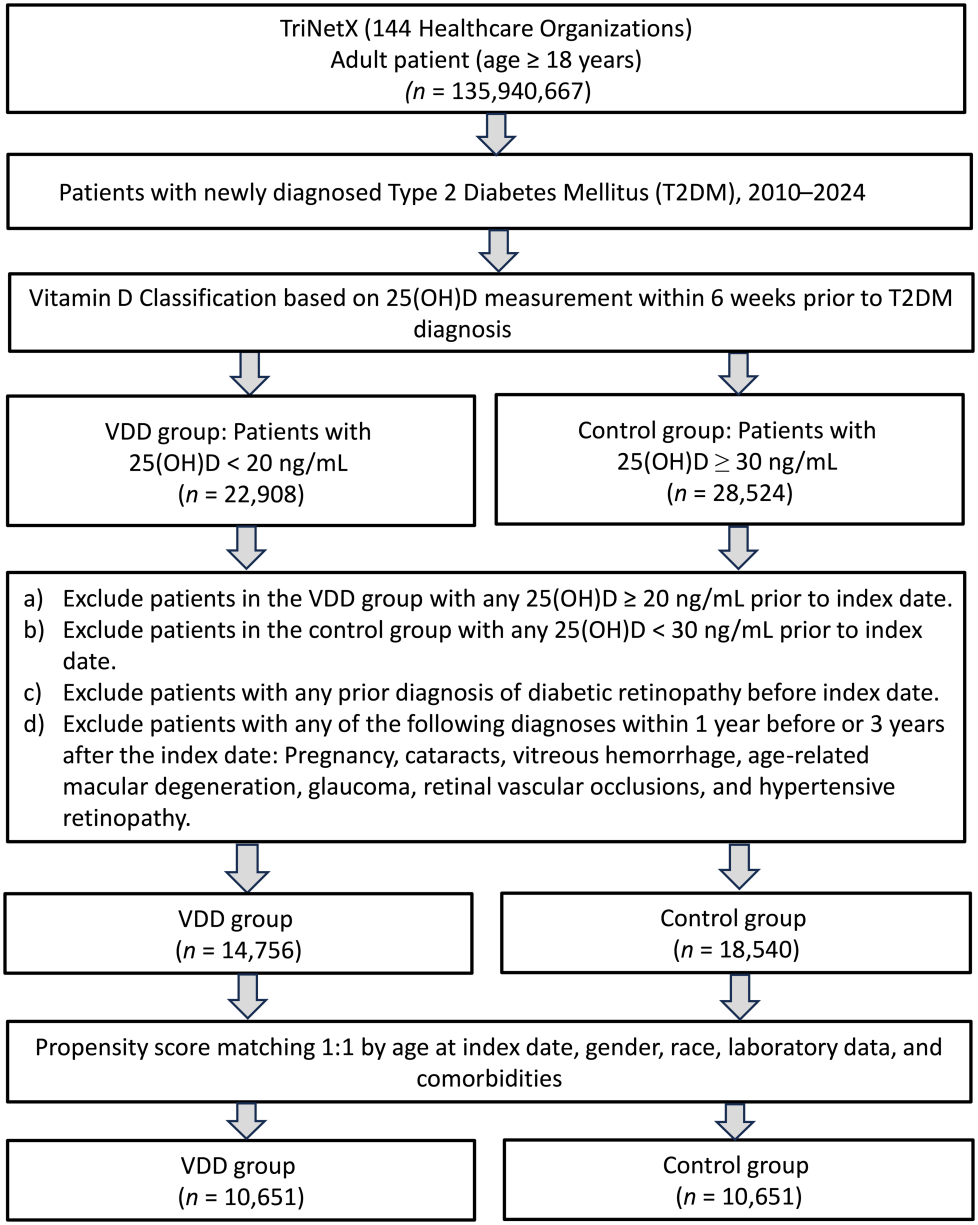
Patient selection from the TriNetx database. VDD, vitamin D deficiency.

As illustrated in Figure 1, our initial screening identified patients who were newly diagnosed with T2DM between January 2020 and December 2022. After assessing vitamin D levels, 14,756 patients were classified into the VDD group (serum 25(OH)D <20 ng/mL), while 18,540 patients with sufficient vitamin D levels (≥30 ng/mL) formed the control group. After propensity score matching, 10,651 patients remained in each group.

**Figure 2:**
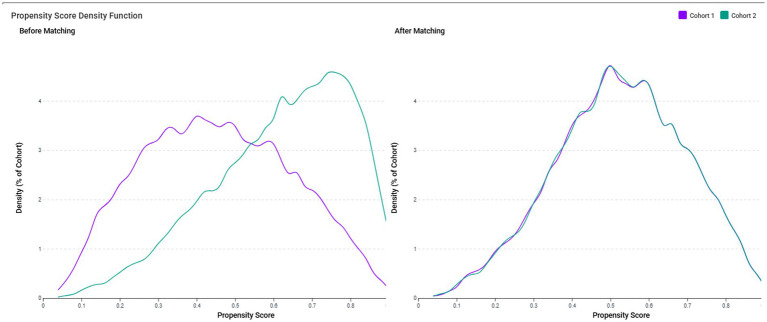
The propensity score density function plots demonstrate the distribution of propensity scores in the vitamin D deficiency (VDD) group (Cohort 1, purple line) and the control group (Cohort 2, green line). Before matching (left panel), the VDD group showed a left-skewed distribution with propensity scores concentrated between 0.2–0.6, while the control group exhibited a right-skewed distribution with propensity scores primarily between 0.4–0.8, indicating substantial differences in baseline characteristics. After 1:1 propensity score matching (right panel), the density functions of both cohorts closely overlapped across the entire range of propensity scores, confirming balanced distribution of baseline characteristics between the matched groups.

Prior to matching, the propensity score density function plots demonstrated significant differences in the distribution of baseline characteristics between the VDD and control groups (Figure 2, left panel). The VDD group (Cohort 1, purple line) showed a left-skewed distribution with propensity scores concentrated between 0.2–0.6, while the control group (Cohort 2, green line) exhibited a right-skewed distribution with propensity scores primarily between 0.4–0.8. This divergence confirmed the substantial differences in baseline characteristics between the groups.

After 1:1 propensity score matching (Figure 2, right panel), the density functions of both cohorts closely overlapped across the entire range of propensity scores, with nearly identical distributions between propensity scores of 0.1–0.8. This visual confirmation of a balanced propensity score distribution complements quantitative assessment using standardized mean differences (SMD <0.1) and validates the effectiveness of our matching procedure in creating comparable groups for outcome analysis. The matched cohorts had a mean age of approximately 56 years, with a female predominance (approximately 58%) (Table 1).

**Table 1:** Baseline characteristics of patients with type 2 diabetes mellitus before and after propensity score matching.

Variables	Before matching			After matching		
	VDD group (*n* = 14,756)	Control group (*n* = 18,540)	SMD^a^	VDD group (*n* = 10,651)	Control group (*n* = 10,651)	SMD^a^
Patient characteristics						
Age at index (years)	52.5 ± 16.1	61.8 ± 14.4	0.611	56.6 ± 14.8	56.5 ± 14.5	0.007
BMI ≥30 (kg/m^2^)	7,162 (48.5%)	7,550 (40.7%)	0.158	4,843 (45.5%)	4,893 (45.9%)	0.009
Female	8,200 (55.6%)	11,489 (62.0%)	0.130	6,157 (57.8%)	6,215 (58.4%)	0.011
White	7,045 (47.7%)	12,686 (68.4%)	0.429	6,074 (57.0%)	6,110 (57.4%)	0.007
Comorbidities/medication						
Essential (primary) hypertension	5,593 (37.9%)	8,803 (47.5%)	0.195	4,389 (41.2%)	4,404 (41.3%)	0.003
Overweight and obesity	4,292 (29.1%)	3,892 (21.0%)	0.188	2,628 (24.7%)	2,673 (25.1%)	0.010
Hyperlipidemia, unspecified	2,693 (18.3%)	5,524 (29.8%)	0.273	2,337 (21.9%)	2,287 (21.5%)	0.011
Neoplasms	2,219 (15.0%)	3,953 (21.3%)	0.163	1847 (17.3%)	1818 (17.1%)	0.007
Chronic kidney disease (CKD)	1,544 (10.5%)	2,632 (14.2%)	0.114	1,267 (11.9%)	1,202 (11.3%)	0.019
Ischemic heart diseases	1,183 (8.0%)	1884 (10.2%)	0.075	951 (8.9%)	928 (8.7%)	0.008
Diseases of liver	1,269 (8.6%)	1,492 (8.0%)	0.020	912 (8.6%)	910 (8.5%)	0.001
Nicotine dependence	1,313 (8.9%)	1,044 (5.6%)	0.126	792 (7.4%)	790 (7.4%)	0.001
Heart failure	947 (6.4%)	1,201 (6.5%)	0.002	689 (6.5%)	660 (6.2%)	0.011
Other chronic obstructive pulmonary disease	630 (4.3%)	927 (5.0%)	0.035	506 (4.8%)	491 (4.6%)	0.007
Cerebrovascular diseases	656 (4.4%)	944 (5.1%)	0.030	497 (4.7%)	521 (4.9%)	0.011
Malnutrition	493 (3.3%)	455 (2.5%)	0.053	322 (3.0%)	310 (2.9%)	0.007
Alcohol related disorders	412 (2.8%)	318 (1.7%)	0.073	248 (2.3%)	241 (2.3%)	0.004
Laboratory data						
Hemoglobin>12	11,023 (74.7%)	14,154 (76.3%)	0.038	8,061 (75.7%)	8,089 (75.9%)	0.006
Albumin g/dL (≥3.5 g/dL)	11,644 (78.9%)	15,782 (85.1%)	0.162	8,668 (81.4%)	8,746 (82.1%)	0.019
Medications						
Antilipemic agents	3,570 (24.2%)	6,729 (36.3%)	0.266	3,059 (28.7%)	3,055 (28.7%)	0.001
ACE inhibitors	2,301 (15.6%)	3,131 (16.9%)	0.035	1761 (16.5%)	1777 (16.7%)	0.004
Angiotensin II inhibitor	1,529 (10.4%)	2,824 (15.2%)	0.146	1,288 (12.1%)	1,288 (12.1%)	0.000

BMI, body mass index; SMD, standardized mean differences; COPD, chronic obstructive pulmonary disease.

a SMD values <0.1 indicate adequate balance between groups.

### 3.2 Primary and secondary outcomes at 1-year and 3-year follow-up

**Figure 3:**
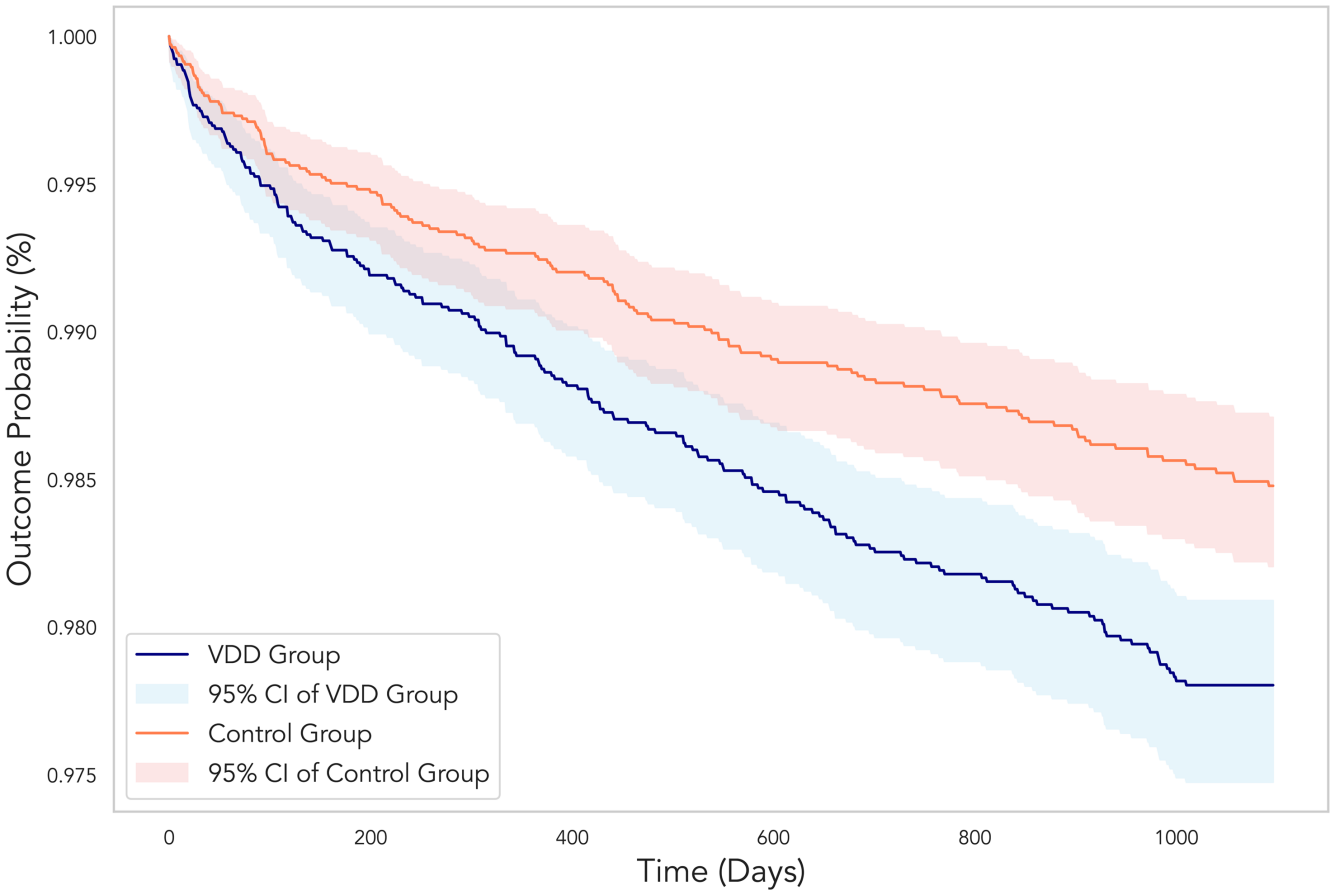
Kaplan–Meier curves showing the cumulative probability of remaining free from diabetic retinopathy over the 3-year follow-up period. The vitamin D deficiency (VDD) group (blue line) demonstrated a significantly higher incidence of diabetic retinopathy compared to the control group (orange line). Shaded areas represent the 95% confidence intervals for each group. The divergence between the two curves illustrates the 45% increased risk of developing diabetic retinopathy in patients with VDD (hazard ratio 1.45, 95% CI 1.17–1.80, *p* < 0.001).

At the 3-year follow-up, patients with VDD showed a significantly higher risk of DR than the vitamin D-sufficient control group (HR 1.45, 95% CI 1.17–1.80, *p* < 0.001) (Table 2 and Figure 3). The VDD group also demonstrated significantly increased risks for secondary outcomes, including hospitalization (HR 1.23, 95% CI 1.17–1.29, *p* < 0.001), emergency department visits (HR 1.17, 95% CI 1.11–1.24, *p* < 0.001), pneumonia (HR 1.18, 95% CI 1.07–1.31, *p* = 0.001), and all-cause mortality (HR 1.51, 95% CI 1.36–1.67, *p* < 0.001) (Table 2).

**Table 2:** Association between vitamin D deficiency and 3-year outcomes in patients with newly diagnosed type 2 diabetes mellitus.

Outcomes	VDD group (*n* = 10,651)	Control group (*n* = 10,651)	HR (95% CI)	*p*-value
	Events (%)	Events (%)		
Diabetic retinopathy	196 (1.84%)	141 (1.32%)	1.45 (1.17–1.80)	<0.001
Hospitalization	3,259 (30.6%)	2,835 (26.6%)	1.23 (1.17–1.29)	<0.001
Emergency department visits	2,987 (28.0%)	2,728 (25.6%)	1.17 (1.11–1.24)	<0.001
Pneumonia	832 (7.8%)	734 (6.9%)	1.18 (1.07–1.31)	0.001
All-cause mortality	911 (8.6%)	631 (5.9%)	1.51 (1.36–1.67)	<0.001

HR, hazard ratio; CI, confidence interval; VDD, vitamin D deficiency.

Similarly, at the 1-year follow-up (Table 3), VDD was associated with an increased risk of DR (HR 1.45, 95% CI 1.11–1.90, *p* = 0.008), hospitalization (HR 1.28, 95% CI 1.21–1.36, *p* < 0.001), emergency department visits (HR 1.26, 95% CI 1.18–1.34, *p* < 0.001), pneumonia (HR 1.16, 95% CI 1.03–1.31, *p* = 0.015), and mortality (HR 1.58, 95% CI 1.40–1.79, *p* < 0.001).

**Table 3:** Association between vitamin D deficiency and 1-year outcomes in patients with newly diagnosed type 2 diabetes mellitus.

Outcomes	VDD group (*n* = 12,007)	Control group (*n* = 12,007)	HR (95% CI)	*p*-value
	Events (%)	Events (%)		
Diabetic retinopathy	123 (1.0%)	88 (0.7%)	1.45 (1.11–1.90)	0.008
Hospitalization	2,739 (22.8%)	2,260 (18.8%)	1.28 (1.21–1.36)	<0.001
Emergency department visits	2,109 (17.6%)	1,769 (14.7%)	1.26 (1.18–1.34)	<0.001
Pneumonia	568 (4.7%)	506 (4.2%)	1.16 (1.03–1.31)	0.015
All-cause mortality	624 (5.2%)	408 (3.4%)	1.58 (1.40–1.79)	<0.001

HR, hazard ratio; CI, confidence interval; VDD, vitamin D deficiency.

### 3.3 Sensitivity analysis of 3-year outcomes

Three sensitivity analyses were conducted to evaluate the robustness of the results (Table 4). In Model I, which included only patients who survived the 3-year follow-up period, the association between VDD and adverse outcomes remained significant. VDD was associated with an increased risk of DR (HR 1.43, 95% CI 1.16–1.76, *p* < 0.001), hospitalization (HR 1.23, 95% CI 1.17–1.30, *p* < 0.001), emergency department visits (HR 1.12, 95% CI 1.10–1.18, *p* < 0.001), and pneumonia (HR 1.20, 95% CI 1.08–1.33, *p* < 0.001).

**Table 4:** Sensitivity analyses of the association between vitamin D deficiency and 3-year outcomes in patients with newly diagnosed type 2 diabetes mellitus.

Outcomes	Model I		Model II		Model III	
	HR (95% CI)	*p*-values	HR (95% CI)	*p*-values	HR (95% CI)	*p*-values
Diabetic retinopathy	1.43 (1.16–1.76)	<0.001	1.79 (1.02–3.13)	0.039	1.26 (1.01–1.57)	0.037
Hospitalization	1.23 (1.17–1.30)	<0.001	1.34 (1.29–1.39)	<0.001	1.28 (1.21–1.35)	<0.001
Emergency department visits	1.12 (1.10–1.18)	<0.001	1.28 (1.23–1.33)	<0.001	1.21 (1.14–1.28)	<0.001
Pneumonia	1.20 (1.08–1.33)	<0.001	1.24 (1.16–1.33)	<0.001	1.21 (1.10–1.34)	<0.001
All-cause mortality	—	—	1.74 (1.64–1.83)	<0.001	1.44 (1.31–1.59)	<0.001

HR, hazard ratio; CI, confidence interval.

Model I: Analysis restricted to patients who survived the entire 3-year follow-up period.

Model II: Analysis included all patients with type 2 diabetes mellitus, regardless of diagnosis timing, with anti-diabetic medication use incorporated into propensity score matching.

Model III: Analysis restricted to patients aged ≥50 years.

In Model II, which expanded the cohort to include all patients with T2DM (not limited to newly diagnosed cases) and incorporated antidiabetic medication use in propensity score matching, the associations between VDD and adverse outcomes were even stronger. VDD was associated with an elevated risk of DR (HR 1.79, 95% CI 1.02–3.13, *p* = 0.039), hospitalization (HR 1.34, 95% CI 1.29–1.39, *p* < 0.001), emergency department visits (HR 1.28, 95% CI 1.23–1.33, *p* < 0.001), pneumonia (HR 1.24, 95% CI 1.16–1.33, *p* < 0.001), and mortality (HR 1.74, 95% CI 1.64–1.83, *p* < 0.001). In addition, we performed a third sensitivity analysis (Model III) restricted to patients aged ≥50 years. As shown in Table 4, VDD remained significantly associated with increased risks of DR, hospitalization, emergency department visits, pneumonia, and all-cause mortality in this subgroup, supporting the robustness of our findings across age groups.

### 3.4 Subgroup analysis of 3-year outcomes by sex

When stratified by sex (Table 5), the association between VDD and DR was significant among female patients (HR 1.41, 95% CI 1.07–1.86, *p* = 0.015) but not among male patients (HR 1.11, 95% CI 0.80–1.53, *p* = 0.55). Both sexes showed significantly increased risks of hospitalization (males: HR 1.28; females: HR 1.24) and emergency department visits (males: HR 1.21; females: HR 1.17). Interestingly, the association between VDD and pneumonia was significant only in females (HR 1.26, 95% CI 1.11–1.42, *p* < 0.001) and not in males (HR 0.99, 95% CI 0.86–1.15, *p* = 0.941). All-cause mortality risk was elevated in both sexes but was more pronounced in females (HR 1.57, 95% CI 1.37–1.80, *p* < 0.001) than in males (HR 1.34, 95% CI 1.16–1.55, *p* < 0.001).

**Table 5:** Analyses of the association between vitamin D deficiency and 3-year outcomes in patients with newly diagnosed type 2 diabetes mellitus by sex.

Outcomes	Male (*n* = 4,100 per group)		Female (*n* = 7,048 per group)	
	HR (95% CI)	*p*-values	HR (95% CI)	*p*-values
Diabetic retinopathy	1.11 (0.80–1.53)	0.55	1.41 (1.07–1.86)	0.015
Hospitalization	1.28 (1.19–1.39)	<0.001	1.24 (1.16–1.32)	<0.001
Emergency department visits	1.21 (1.11–1.32)	<0.001	1.17 (1.10–1.24)	<0.001
Pneumonia	0.99 (0.86–1.15)	0.941	1.26 (1.11–1.42)	<0.001
All-cause mortality	1.34 (1.16–1.55)	<0.001	1.57 (1.37–1.80)	<0.001

HR, hazard ratio; CI, confidence interval.

### 3.5 Association between vitamin D insufficiency and 3-year outcomes

In an exploratory analysis examining patients with vitamin D insufficiency (25(OH)D levels between 20–30 ng/mL) compared to vitamin D-sufficient controls (Table 6), no significant association was observed for DR (HR 1.00, 95% CI 0.82–1.23, *p* = 0.976). However, vitamin D insufficiency was associated with mild but statistically significant increases in the risk of hospitalization (HR 1.09, 95% CI 1.04–1.14, *p* < 0.001), emergency department visits (HR 1.06, 95% CI 1.01–1.11, *p* = 0.025), pneumonia (HR 1.13, 95% CI 1.03–1.24, *p* = 0.014), and all-cause mortality (HR 1.16, 95% CI 1.05–1.28, *p* = 0.003). These associations, although significant, were considerably weaker than those observed for VDD, suggesting a potential dose–response relationship between vitamin D status and adverse outcomes in patients with T2DM.

**Table 6:** Association between vitamin D insufficiency and 3-year outcomes in patients with newly diagnosed type 2 diabetes mellitus.

Outcomes	VDI group (*n* = 12,925)	Control group (*n* = 12,925)	HR (95% CI)	*p*-value
	Events (%)	Events (%)		
Diabetic retinopathy	187 (1.4%)	189 (1.5%)	1.00 (0.82–1.23)	0.976
Hospitalization	3,485 (27.0%)	3,298 (25.5%)	1.09 (1.04–1.14)	<0.001
Emergency department visits	3,366 (26.0%)	3,259 (25.2%)	1.06 (1.01–1.11)	0.025
Pneumonia	930 (7.2%)	840 (6.5%)	1.13 (1.03–1.24)	0.014
All-cause mortality	834 (6.5%)	729 (5.6%)	1.16 (1.05–1.28)	0.003

HR, hazard ratio; CI, confidence interval; VDI, vitamin D insufficiency.

## 4 Discussion

This retrospective cohort study revealed a significant association between VDD and DR in patients with newly diagnosed T2DM. Patients with VDD had a 45% higher risk of developing retinopathy over 3 years than those with sufficient vitamin D levels. This association remained consistent in both the 1-year and 3-year follow-up analyses. Sensitivity analyses, including one restricted to survivors and another incorporating anti-diabetic medication use, confirmed the robustness of the findings. Sex-stratified analysis showed that the association was primarily significant in female patients. Notably, patients with vitamin D insufficiency did not show an increased risk, suggesting a threshold effect. Beyond retinopathy, VDD has also been linked to higher risks of hospitalization, emergency department visits, pneumonia, and mortality, highlighting its potential as a marker of the overall health status of patients with diabetes.

Our findings revealed a robust association between VDD at the time of T2DM diagnosis and the subsequent development of DR. The novelty of this study lies in several key aspects. First, we specifically focused on patients with newly diagnosed T2DM, allowing us to examine the impact of baseline vitamin D status on long-term diabetes complications and treatments. This approach minimizes potential reverse causality, whereby diabetic complications may lead to reduced outdoor activity and consequently lower vitamin D levels (18, 19). Second, our study employed rigorous propensity score matching to balance a comprehensive set of confounding variables to enable more reliable estimates of the association between vitamin D status and diabetic outcomes. Third, our dose-response analysis comparing VDD (<20 ng/mL) and insufficiency (20–30 ng/mL) against sufficiency (≥30 ng/mL) suggests a threshold effect, where the risk of DR significantly increases only when vitamin D levels fall below 20 ng/mL. This finding has important clinical implications and suggests that maintaining vitamin D levels above the deficiency threshold may mitigate the risk of DR. The biological mechanisms potentially mediating this association include anti-inflammatory, antioxidant, and anti-angiogenic properties (20–24). Vitamin D receptors are expressed in retinal tissues, and experimental studies have shown that vitamin D can inhibit VEGF-induced endothelial cell proliferation and angiogenesis (24). Additionally, vitamin D may regulate retinal microvascular integrity by modulating inflammatory pathways and reducing oxidative stress, which are key factors in DR pathogenesis (2, 25).

While our analysis focused on VDD, it is important to consider that DR is a multifactorial condition. Hypertension, dyslipidemia, and chronic kidney disease are among the established comorbidities that independently elevate the risk of developing DR (26–28). Although our propensity score matching balanced these conditions between the VDD and control groups, their complex interactions and varying severities may not have been fully captured in our analysis. Additionally, the duration and control of these comorbidities prior to diabetes diagnosis could influence baseline retinal health. While we adjusted for these factors as categorical variables, residual confounding from disease severity or duration remained possible.

A recent meta-analysis by Petrea et al. (6) found a significant association between lower vitamin D levels and increased odds of DR (OR, 1.17). However, most prior studies in that meta-analysis (6) employed cross-sectional designs, which precluded the establishment of temporal relationships between VDD and retinopathy development (29). Furthermore, previous studies in that meta-analysis (6) often included heterogeneous diabetes populations with varying disease durations, making it difficult to distinguish the effects of VDD from those of long-standing diabetes complications (6, 30). Notably, many previous studies had limited adjustments for confounding factors and relatively small sample sizes, limiting their statistical power and generalizability (31, 32). Our large sample size from a multi-institutional database enhances both the precision of our estimates and their applicability to diverse clinical settings. Finally, only a few studies have examined sex-specific differences in the association between VDD and DR. Our stratified analysis revealed stronger associations among females contributing valuable insights into potential effect modification by sex, which may inform personalized preventive strategies.

The sex-specific association between VDD and DR requires consideration of several biological mechanisms. Sex hormones, particularly estrogen, may provide vascular protection through their antioxidant effects (33). Sex-based differences in vitamin D metabolism and vitamin D receptor expression might make females more responsive to the vascular protective effects of vitamin D. Additionally, fundamental sex differences in immune responses, adiposity distribution, insulin sensitivity, and genetic factors affecting the vitamin D pathway could contribute to the observed sex-specific vulnerability to DR in vitamin D-deficient states (34). These mechanisms warrant further investigation in future targeted studies.

Our study found that VDD was associated with a 23% increased risk of hospitalization and a 17% increased risk of emergency department visits over 3 years. These associations remained consistent in the 1-year analysis and across the sensitivity analyses. Increased healthcare utilization in patients with VDD may reflect several underlying mechanisms. VDD may serve as a marker of poor overall health status and behavior, including reduced physical activity, poor nutrition, and suboptimal self-care, which can contribute to diabetes complications requiring acute care (35, 36). Additionally, vitamin D has immunomodulatory effects that may influence susceptibility to infections and inflammatory conditions, which are common reasons for hospitalization in patients with diabetes (37). The economic implications of these findings are significant. Hospitalizations and emergency department visits represent significant healthcare costs in diabetes management (38–40). If vitamin D supplementation in deficient patients could reduce these events, it might offer a cost-effective intervention to improve outcomes and reduce healthcare expenditures.

Our finding of an 18% increased risk of pneumonia in vitamin D-deficient patients with newly diagnosed T2DM is consistent with emerging evidence regarding the role of vitamin D in immune function and respiratory health. This association remained significant in both 1-year and 3-year analyses, with hazard ratios of 1.16 and 1.18, respectively. Interestingly, our sex-stratified analysis revealed that the association between VDD and pneumonia was significant only in females (HR 1.26) and not in males (HR 0.99). This sex-specific effect mirrors our findings on DR and suggests potential biological differences in vitamin D metabolism (41) or immune responses between the sexes (42). Previous studies have reported an association between VDD and an increased risk of respiratory infections, including pneumonia (37). A meta-analysis of individual participant data from 25 randomized controlled trials found that vitamin D supplementation reduced the risk of acute respiratory infections, with greater benefits in those with baseline VDD (43). Our results extend these findings specifically to patients with newly diagnosed type 2 diabetes, a population with increased susceptibility to infections due to immune dysfunction.

Perhaps the most striking finding in our study is the 51% increased risk of all-cause mortality over 3 years associated with VDD in patients with newly diagnosed T2DM. The association was even stronger in the 1-year analysis (HR, 1.58), suggesting that VDD may be particularly predictive of short-term mortality risk. Our sex-stratified analysis revealed that while mortality risk was elevated in both sexes, the association was stronger in females (HR 1.57) than in males (HR 1.34), consistent with our other findings of sex-specific effects. Several mechanisms may explain this association. Vitamin D has direct cardiovascular effects, including regulation of the renin-angiotensin system, endothelial function, and vascular smooth muscle cell proliferation (44, 45). VDD may contribute to cardiovascular events, which are the leading cause of death in patients with diabetes.

Our findings have several important clinical implications. For newly diagnosed T2DM patients, vitamin D status assessment could serve as a simple, cost-effective screening tool for identifying those at a higher risk of developing DR and other complications. Based on the threshold effect observed, maintaining serum 25(OH)D levels above 20 ng/mL may be sufficient to reduce DR risk, particularly in female patients. Healthcare providers might consider more vigilant retinal monitoring in vitamin D-deficient patients, especially females, with early ophthalmologic referral and more frequent retinal examinations during the first 3 years after diagnosis. Although randomized controlled trials are needed to establish definitive supplementation guidelines, our results suggest that correcting vitamin D deficiency in patients newly diagnosed with T2DM might represent a modifiable intervention to potentially reduce diabetic complications. The sex-specific associations also highlight the importance of personalized approaches for both risk assessment and management strategies.

Our study has several limitations that should be acknowledged. First, residual confounding factors cannot be entirely ruled out in observational studies. Unmeasured factors, such as physical activity, sun exposure, dietary habits, and vitamin D supplementation, may influence both vitamin D levels and diabetes outcomes (46). Additionally, missing data for certain laboratory parameters (e.g., hemoglobin and albumin) were addressed through complete-case analysis during propensity score matching, which excluded patients with missing values for these variables. While this ensured that matching was based on actual observed data, it may have introduced a selection bias or limited the generalizability of our findings. Second, the study relied on a single 25(OH)D measurement within 6 weeks prior to T2DM diagnosis, which may not fully represent the long-term vitamin D status. Seasonal fluctuations could influence serum levels; however, adjustment for seasonality was not feasible within the TriNetX platform because of the unavailability of the test date and geographic data. Although we performed a sensitivity analysis using patients with multiple vitamin D measurements, the resulting sample size was markedly reduced, limiting the statistical power. This limitation should be considered when interpreting our findings. Third, while we focused on newly diagnosed type 2 diabetes to minimize reverse causality, some patients might have had undiagnosed diabetes for varying periods before their formal diagnosis (47), potentially influencing baseline vitamin D levels and complication risks. Fourth, the ascertainment of DR and other outcomes relied on diagnostic codes in electronic health records, which may lack sensitivity for detecting early or mild cases and could be subject to coding errors or variations in clinical practice. Fifth, our database primarily included patients from the United States, which may limit the generalizability of our findings to populations with different genetic backgrounds, healthcare systems, and sunlight exposure patterns. Furthermore, although race was recorded in our dataset, the sample sizes of several racial subgroups were insufficient to perform statistically reliable stratified analyses. Finally, our study design could not establish causality between VDD and diabetes outcomes. Although our findings suggest associations, randomized controlled trials of vitamin D supplementation are needed to determine whether correcting VDD can reduce the risk of complications in patients with newly diagnosed diabetes.

## 5 Conclusion

In newly diagnosed T2DM, VDD was independently associated with increased risks of DR, hospitalization, emergency department visits, pneumonia, and all-cause mortality over a three-year follow-up period. These associations are particularly pronounced in female patients and appear to follow a threshold effect, with significantly elevated risks observed primarily in vitamin D-deficient rather than insufficient individuals. Our findings suggest that assessment of vitamin D status may be valuable for risk stratification in newly diagnosed T2DM, and addressing VDD may represent a modifiable risk factor for improving outcomes. Large-scale randomized controlled trials are warranted to determine whether vitamin D supplementation can effectively reduce the burden of diabetic complications and mortality in this population.

## Data Availability

The raw data supporting the conclusions of this article will be made available by the authors, without undue reservation.
